# Bilateral multiple periprosthetic hip fractures and joint dislocations secondary to general convulsive seizures

**DOI:** 10.1186/s12891-021-04557-2

**Published:** 2021-08-09

**Authors:** Qingyu Zhang, Fuqiang Gao, Wei Sun, Zirong Li

**Affiliations:** 1 Department of Orthopedics, Shandong Provincial Hospital Affiliated to Shandong First Medical University, No.324, Road Jing Wu Wei Qi, Jinan, 250021 Shandong China; 2 Department of Orthopedics, China-Japan Friendship Hospital, 2 Yinghuadong Road, Chaoyang District, Beijing, 100029 China

**Keywords:** Seizure, Total hip arthroplasty, Periprosthetic joint fracture, Dislocation, Reconstruction, Case report

## Abstract

**Background:**

During a seizure, there is a powerful and forceful contraction of muscles which may lead to fractures or joint dislocations. However, multiple periprosthetic hip fractures and joint dislocations secondary to seizures have not been reported.

**Case presentation:**

A 49-year-old male developed spontaneous and bilateral multiple periprosthetic hip fractures and joint dislocations (including displaced fracture of the proximal right femur, avulsion fracture of the left lesser trochanter, left acetabular fracture and bilateral joint dislocations) secondary to generalized convulsive seizures which occurred within few hours after bilateral total hip arthroplasties (THAs). Bilateral open reconstruction and fixation were performed on the 21st day after primary THAs and on 2-year follow-up, the patient showed satisfactory functional outcome.

**Conclusions:**

Multiple periprosthetic hip fractures and joint dislocations secondary to seizure are extremely rare, and treatment targets for these injuries should focus on fracture healing and limb function recovery. Craniocerebral operation could bring an elevated risk of seizure; meanwhile, subsequent corticosteroid replacement threapy was complicated by secondary osteoporosis. Therefore, anti-osteoporotic and anti-epileptic therapy should be considered in this type of patients to avoid fracture and dislocation after arthroplasty.

## Background

Although aseptic loosening, instability, osteolysis and infection are common reasons for revision after total hip arthroplasty (THA), malfunction of endoprostheses also can be caused by periprosthetic fracture (PPF) [1]. Patients who have epileptic seizures are at an increased risk of sustaining accidental injuries including burns, contusions, and head injury [2, 3]. During a convulsion, there is an uncontrolled powerful contraction of all of the muscles which may lead to fracture or dislocation, with an incidence being about 1.1% [3]. There is a handful of literature describing seizure-induced fracture of vertebrae, femoral neck, proximal femur, and acetabulum as well as dislocation of shoulder joints [4, 5] but currently, only few cases of periprosthetic acetabular fractures secondary to seizures were reported [6, 7].

Here we present a case of simultaneously bilateral multiple periprosthetic hip fractures and joint dislocations (including displaced fracture of the proximal right femur, avulsion fracture of the left lesser trochanter, left acetabular fracture and bilateral joint dislocations) in a 49-year-old male following a generalized convulsive seizure in view of its rarity and favorable prognosis after appropriate management. Meanwhile, we also described the occurrence mechanism of these injuries, as well as the experience and deficiencies in the treatment of this patient. It may increase the awareness of such injuries among emergency physicians, neurologists, and orthopedic surgeons. We present the following article in accordance with the CARE reporting checklist.

## Case presentation

**Fig. 1:**
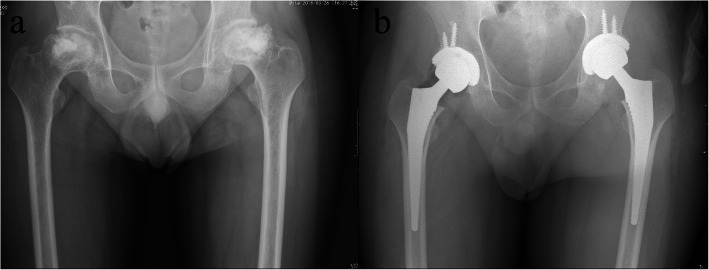
The anteroposterior radiographs of bilateral hip joints before (**a**) and after (**b**) bilateral total hip arthroplasties. **a** Radiograph taken 2 days before THA showed bilateral femoral head collapse and secondary osteoarthritis, which indicated ARCO IV stage osteonecrosis of the femoral head; (**b**) radiograph taken immediately after bilateral THA showed satisfactory location and fixation of bilateral femoral and acetabular prostheses

A 49-year-old male was referred to the orthopedic department of a tertiary hospital with pain in both hips for 19 months and the X-ray examination showed bilateral femoral head collapse and secondary osteoarthritis (ARCO stage IV osteonecrosis of the femoral head, ONFH) (Fig. 1a). He underwent craniocerebral surgery for craniopharyngioma approximately 2 years ago and had a long-term history of prednisolone administration (5 mg orally, twice daily for 2 yrs.). This patient received bilateral light bulb surgeries (removal of necrotic bone and impaction of artificial bone through a window at the femoral head-neck junction) 16 months ago and after that, hip pain was relieved for several months but aggravated 6 months ago. Considering his good physical condition, one-stage bilateral total hip arthroplasties were performed through a posterior approach. Two cementless press-fit endoprostheses were implanted on both sides using 52-mm PINNACLE cups with POROCOAT porous coating (Depuy, Warsaw, IN) and polyethylene liners with size 11 CORAIL stems (Depuy) with 36-mm ceramic heads (Fig. 1b).

Two hours after returning to the general ward, the patient suddenly had violent limb twitching and loss of consciousness which spontaneously relieved within 1 min. We consulted the neurology department, and then performed cerebral MRI and electroencephalogram (EEG). Before any further intervention was applied, another episode of generalized convulsive seizure with severe extremity contractions occurred 4 h after the first one. There was no evidence of open wound, falling from bed, external trauma, bruising or distal neurovascular deficits during seizure episode and afterward. The patient was not a known epileptic before.

**Fig. 2:**
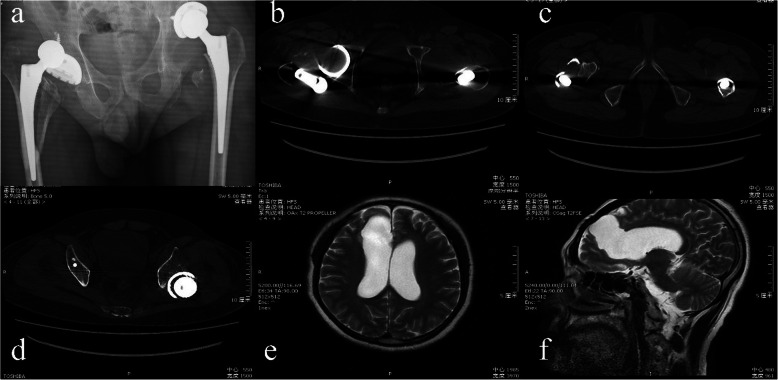
The anterior-posterior radiograph of the bilateral hip (**a**) showed dislocation of the right femoral head, displaced oblique fracture of the right proximal femur involving greater trochanter, upward dislocation of the left hip joint involving acetabular endoprosthesis components, fracture of the right acetabular and avulsion fracture of right lesser trochanter; computed tomography scan showed upward and backward dislocation of the right femoral head (**b**), displaced oblique fracture of the right proximal femur involving greater trochanter (**c**) and upward dislocation of the left hip joint involving acetabular endoprosthesis components (**d**); MRI showed a large encephalomalacia focus (4 cm × 3.5 cm × 3 cm size) located at the right frontal lobe and communicating with the lateral ventricle (**e**, **f**)

Associated symptoms being controlled, this patient complained of severe pain and immobility of both hips. Meanwhile, both lower limbs were externally rotated and markedly foreshortened on clinical examination. Plain radiographs and CT of the hip joints showed backward and upward dislocation of the right femoral head, displaced oblique fracture of the proximal right femur involving greater trochanter, upward dislocation of the left hip joint involving acetabular endoprosthesis components, fracture of the left acetabular and avulsion fracture of left lesser trochanter (Fig. 2 a, b, c & d). Cerebral MRI demonstrated a large encephalomalacia focus (4 cm × 3.5 cm × 3 cm size) located at the right frontal lobe and communicating with the lateral ventricle (Fig. 2 e & f).

Since the patient was vulnerable after the last operation, a bilateral revision of total hip arthroplasty was performed in one session on the 21st day after THA when the patient’s condition has stabilized, with anti-epileptic drugs and prednisolone continuing to be administered before revision. The posterolateral approach was followed on both sides. The right hip surgery was performed first: after removal of the femoral prosthesis, fracture flats of the proximal femur were restored and fastened with three titanium cables; then a new set of non-cemented long-stem prosthesis (DePuy) and 36-mm ceramic head was installed, and the dislocated hip joint was reducted. As to the left hip joint, since the superior acetabular bone was defective, an allogeneic femoral head was trimmed and fixed to the region of bone loss by using three cannulated screws. Acetabulum being carefully polished, a new porous 54-mm PINNACLE acetabular cup (Depuy) was placed and fixed with 4 cancellous bone screws; then a 36-mm highly cross-linked polyethylene lining was embedded and dislocated femoral head was reducted.

**Fig. 3:**
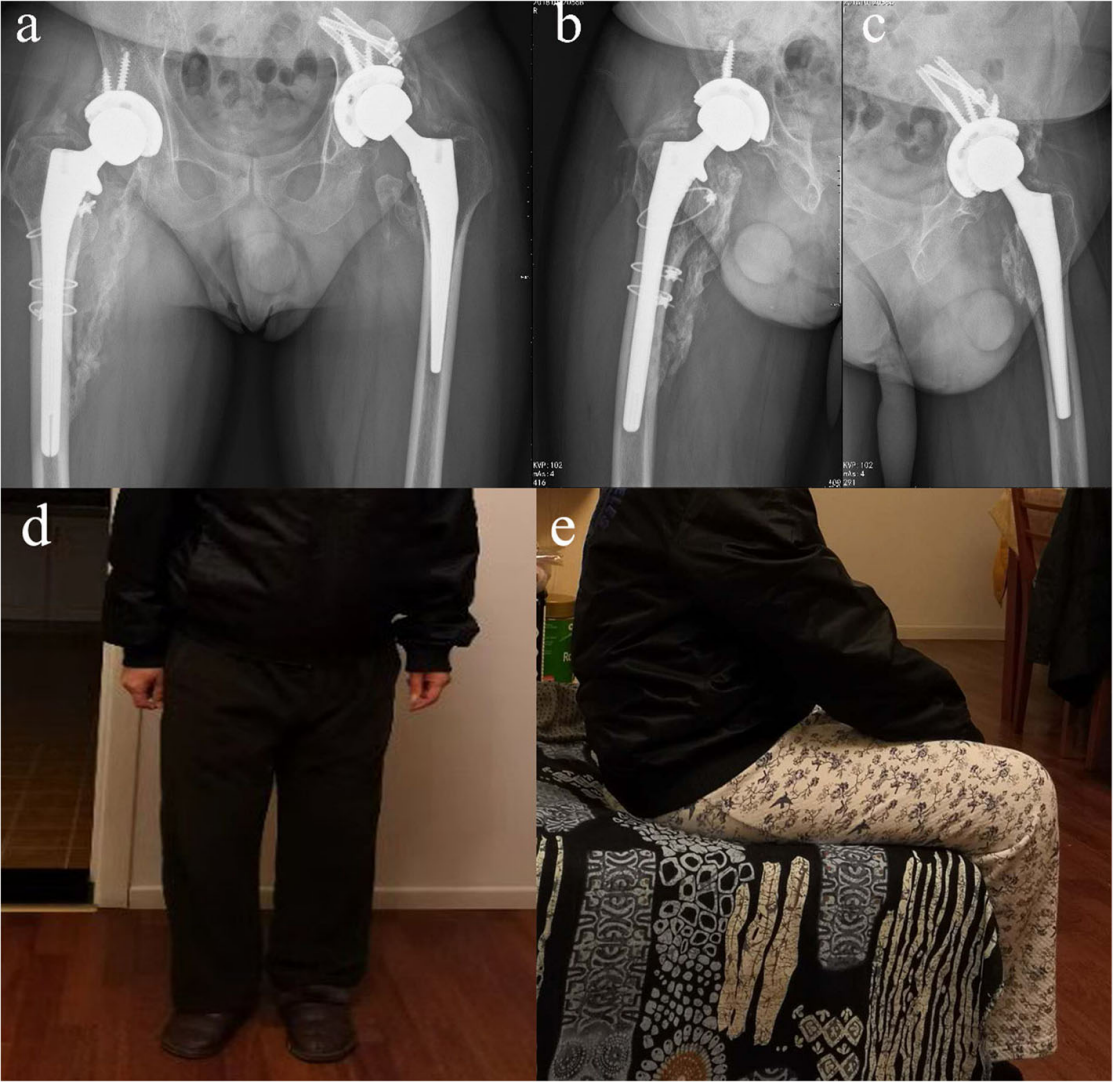
The anterior-posterior (**a**) and lateral (**b** & **c**) radiograph of bilateral hip joints, which taken at 6th month after bilateral THA revision, showed the good position of bilateral prostheses, healing of bone graft on the right side and bilateral ossifying myositis around hip joints. The patient restored a partial range of motion of injured hip joints at the 6th month after bilateral revision

After the operation, the patient was shifted to the intensive care unit and closely monitored for the first 24 h. The postoperative practices including straight-leg elevation, ankle pump and quadriceps isometric contraction were started on the 1st day after surgery and the patient began progressive bedside standing and ambulating.2 weeks later. On 6-month follow-up, there was a good union of fractures both clinically and radiologically (Fig. 3a, b & c); On 2-year follow-up, the patient was pain-free, capable of full weight-bearing walking without support (Fig. 3d & e). Since continuing anti-epileptic therapy, the patient has experienced no further seizures.

## Discussion and conclusions

Periprosthetic fracture is one of the toughest problems after total hip arthroplasty, which often occurs following a high energy trauma or secondary to any indirect mechanisms that weaken surrounding bones like osteomalacia and osteoporosis [8, 9]. Injuries of the case described in the current paper were caused by two successive episodes of epilepsy occurring within a short time after THA and the patient presented with periprosthetic femoral fracture (PFF), periprosthetic acetabular fracture (PAF) and bilateral joint dislocations simultaneously. This kind of multiple injuries has never been introduced in former literature. The unique occurrence mechanism, operation plan and some lessons learned during perioperative treatment were described and discussed in this article.

Postoperative PFF, which generally happens following a low-energy fall from standing height, shows a close relationship with stem loosening and poor clinical outcomes [10, 11]. Bone density, fixation mode (cemented or non-cemented), and stem design are all important factors influencing the probability of such fractures [12]. The treatment algorithm of PFF should follow the Vancouver classification [13]. In this case, the PFF on the left side was assigned to type AL and since it was a simple avulsion fracture and did not affect the stability of the femoral prosthesis, no operative management was adopted. While on the right side both lesser trochanter and the cortical bone around the femoral component were involved, and prosthetic loosening was identified intraoperatively, therefore this fracture was assigned as Vancouver type B2. Up to now, there were several classification systems of PAF [8, 14–16] and according to the Paprosky classification system, implant stability and timing (intraoperative or postoperative) of fractures are the two crucial prognostic and therapeutic predictors [8]. For this case, the periprosthetic acetabular fracture on the left side should be assigned to type 3b [15]. PAF with stable acetabular components can be treated conservatively; however, for this patient, a massive bone defect was located at the ischia and the inner wall of the acetabulum, and therefore reconstruction options other than large allogeneic bone graft and tantalum components obviously could not provide sufficient support and stability [17, 18].

Dislocation of bilateral joints was due to the powerful and violent contraction of hip muscle during convulsion which generated a force directing proximal femur towards the groin [4, 19]. For this patient, the joint capsule was injured during total hip arthroplasty and wherefore bilateral joint dislocations were brought about [4, 20]. It has been reported that the adoption of the posterolateral approach was associated with an increased probability of backward dislocation after hip arthroplasty, which was in accordance with this case [20, 21]. The lesser trochanter serves as the site of attachment for the iliopsoas complex and avulsion injury of the lesser trochanter on the left side represents the extreme on a continuum of iliopsoas strain contraction [5]. In former literature, lesser trochanter avulsions have been described in young runners or jumpers during vigorous hip flexion [5]. On the other hand, the displaced femoral fracture on the right side may be induced by the powerful and unbalanced antagonism of the internal rotation muscle group and external rotation muscle group of the hip joint.

Some lessons could be learned from this case. Surgical stress and a history of former craniocerebral operation both act as incentives for this epileptic attack. Other possible etiologies for seizures such as hypocalcemia, hypoglycemia and dyselectrolytemia were excluded by using routine biochemical investigations. It was negligence that anti-epileptic therapy was not administrated before arthroplasty; which was partly because the patient had never had an epileptic seizure before. After the first attack of seizure, although associated examinations were performed, anti-epileptic medication was not applied immediately and the occurrence of the second seizure induced this terrible problem. Another negligence was that we did not attempt to determine bone density/osteoporosis prior to the time of surgery considering the patient’s age and body condition. This patient was admitted for glucocorticoid-induced ONFH, which was the most common type of non-traumatic ONFH. Long-term administration of antiepileptic medications and corticosteroid may also cause anticonvulsant osteoporosis and therefore act as a risk factor for epilepsy-induced fracture [6]. There was not an attempt to quantify cortical thinning/osteoporosis or the possibility of a surgical problem that predisposed the patient to post-implant fracture. The history of prolonged corticosteroid use deserves more caution in the treatment of patients with ONFH in the future. In this case, treatment to improve bone density, such as empiric treatment with vitamin D, calcium supplement and possible bisphosphanate should be applied since his reparative surgeries.as prophylaxis. This patient has been informed to test bone density routinely and take anti-osteoporotic medication when needed.

Bilateral periprosthetic hip fractures and joint dislocations induced by seizure are life-threatening injuries. Treatment targets for periprosthetic hip fractures and joint dislocations should focus on fracture healing and limb function recovery. The craniocerebral operation could bring an elevated risk of seizure and subsequent corticosteroid use was associated with secondary osteoporosis. Therefore, anti-osteoporotic and anti-epileptic therapy should be considered in this type of patients to avoid fracture and dislocation after arthroplasty.

## Data Availability

The datasets used and/or analyzed during the current study are available from the corresponding author on reasonable request.

## Abbreviations

THA: Total hip arthroplasty
PPF: Periprosthetic fracture
ONFH: Osteonecrosis of the femoral head
EEG: Electroencephalogram
PFF: Periprosthetic femoral fracture
PFA: Periprosthetic acetabular fracture
